# Commentary on “Are Individuals With Type 2 Diabetes Metabolically Inflexible? A Systematic Review and Meta‐Analysis”

**DOI:** 10.1002/edm2.70068

**Published:** 2025-06-03

**Authors:** Katerina Koudelkova, Cedric Moro, Jan Gojda

**Affiliations:** ^1^ Department of Internal Medicine University Hospital Královské Vinohrady and Third Faculty of Medicine, Charles University Prague Czech Republic; ^2^ I2MC, INSERM, Université de Toulouse Toulouse France

**Keywords:** metabolic flexibility, type 2 diabetes, Δ respiratory exchange ratio

To the Editor,

We read with great interest the recent systematic review examining metabolic flexibility (MetFlex), as assessed by respiratory exchange ratio (ΔRER) during hyperinsulinaemic‐euglycaemic clamp, across lean, overweight/obese, and type 2 diabetes (T2D) groups [1]. We commend the authors for their comprehensive effort and agree that such a synthesis was timely and much needed. However, we believe that several methodological limitations and the conceptual framing of MetFlex in the review warrant further discussion to avoid oversimplification of this physiologically complex phenomenon, and we would like to offer a few reflections and suggestions in that regard.

Firstly, the authors concluded that MetFlex is not linked to the T2D diagnosis but rather to BMI. However, BMI alone is an imprecise measure of adiposity and does not capture important differences in body composition or fat distribution, both of which are highly relevant to metabolic health [2]. For example, individuals with similar BMI can have markedly different proportions of visceral adipose tissue, ectopic lipid deposition, and lean mass [3], all of which can influence MetFlex [4]. The limitations of relying solely on BMI have also been acknowledged in the recently proposed diagnostic criteria for obesity, which emphasise the need to assess body composition and functional impairments rather than BMI thresholds alone [2].

Also, some studies included in the review used BMI matching to compare individuals with type 2 diabetes and those with overweight/obesity [5], while others specifically used patients with overweight/obesity and insulin resistance [6], making it even harder to disentangle the relationship between these two phenomena.

The interpretation of MetFlex in individuals with T2D presented in the review also warrants caution due to several sources of heterogeneity across the included studies. First, as the authors mentioned, the use and type of antidiabetic medication varied widely, ranging from diet‐only management to regimens including metformin, sulfonylureas, insulin, thiazolidinediones, and DPP‐4 inhibitors, often in combination. Notably, medications such as metformin are known to increase insulin sensitivity, which could alter substrate utilisation and obscure differences between T2D and obese individuals without diabetes [7]. The duration of diabetes ranged from as little as 3 months [8] to nearly 30 years [9], spanning distinct stages of disease progression with likely differences in microvascular complications, insulin secretion, and insulin resistance, which all could impact independently on substrate metabolism. These factors critically influence metabolic health and should not be overlooked.

Another important point is that although the authors state that the analysis was adjusted for sex, it is unclear how this was achieved in studies that report only pooled results from small, sex‐heterogeneous samples (e.g., 4 men and 2 women in a group, [10]). Without individual‐level data or sex‐stratified outcomes, true adjustment for sex‐specific differences in MetFlex is not feasible, and this limitation should be explicitly acknowledged. The fact that the T2D group contained 76% of men makes it even harder to truly account for the differences.

In addition, we noted a potential discrepancy in the citation of Faerch, 2011 [11] within the review. While the review refers to this study as involving individuals with T2D, overweight, and lean subjects, the original publication appears to include groups with prediabetes and controls and reports different sample sizes than stated.

The authors correctly state that the glucose clamp model with ΔRER measured by indirect calorimetry is used as the gold standard of measurement of MetFlex, though the validity of this measure has been questioned by a number of other authors [12, 13]. MetFlex is a broader physiological concept that encompasses the capacity of multiple tissues—including skeletal muscle, adipose tissue, and liver—to adapt substrate utilisation to changes in metabolic state (e.g., fasting, feeding, exercise, or insulin stimulation) [14, 15, 16]. It is context‐dependent and regulated by complex interactions involving mitochondrial function, insulin signalling, substrate availability, and hormonal control [17]. The use of ΔRER during hyperinsulinaemic‐euglycaemic clamp, while experimentally convenient and mostly used, captures only a narrow aspect of this adaptability, primarily reflecting skeletal muscle substrate switching upon supraphysiological insulin stimulation. This measure may fail to capture the tissue‐nuanced impairments in MetFlex present across metabolically heterogeneous populations. Moreover, in the review itself, high variability in ΔRER was also observed in the lean group. Taken together, we are inclined to think that clamp derived ΔRER alone may not be a sufficient physiological model to assess MetFlex, and interpreting group differences solely through this lens risks oversimplification.

We highly support the authors' recommendation for more complete, consistent, and transparent reporting of methodology and metabolic outcomes. In addition to ΔRER and more detailed clamp parameters, calorimeter measurement parameters (calibration, post‐calorimetric simulation), gender‐stratified patient data, duration of diabetes, description of medication and its withdrawal, as well as compensation parameters and physical activity status.

In summary, this review provides a valuable basis for discussing more precise methods for measuring MetFlex.

We thank the authors for their contribution to the field.

## Author Contributions

K.K. conceived the idea and prepared the initial draft of the manuscript. J.G. and C.M. provided critical feedback, revised the manuscript for intellectual content, and supervised the development of the final version. All authors read and approved the final manuscript.

## Conflicts of Interest

The authors declare no conflicts of interest.

## Data Availability

The data that support the findings of this study are available from the corresponding author upon reasonable request.
